# Large-scale mitogenomics enables insights into Schizophora (Diptera) radiation and population diversity

**DOI:** 10.1038/srep21762

**Published:** 2016-02-25

**Authors:** Ana Carolina M. Junqueira, Ana Maria L. Azeredo-Espin, Daniel F. Paulo, Marco Antonio T. Marinho, Lynn P. Tomsho, Daniela I. Drautz-Moses, Rikky W. Purbojati, Aakrosh Ratan, Stephan C. Schuster

**Affiliations:** 1 Singapore Centre for Environmental Life Sciences Engineering, Nanyang Technological University, Singapore, 637551; 2 Centro de Biologia Molecular e Engenharia Genética and Departamento de Genética, Evolução e Bioagentes, Universidade Estadual de Campinas, Campinas, SP, 13083-875, Brazil; 3 Faculdade de Filosofia Ciências e Letras de Ribeirão Preto, Departamento de Biologia, Universidade de São Paulo, Ribeirão Preto, SP, 14040-901, Brazil; 4 Center for Comparative Genomics and Bioinformatics, Department of Biochemistry and Molecular Biology, The Pennsylvania State University, University Park, PA, 16802, USA; 5 Department of Public Health Sciences and Center for Public Health Genomics, University of Virginia, Charlottesville, VA, 22908, USA

## Abstract

True flies are insects of the order Diptera and encompass one of the most diverse groups of animals on Earth. Within dipterans, Schizophora represents a recent radiation of insects that was used as a model to develop a pipeline for generating complete mitogenomes using various sequencing platforms and strategies. 91 mitogenomes from 32 different species were sequenced and assembled with high fidelity, using amplicon, whole genome shotgun or single molecule sequencing approaches. Based on the novel mitogenomes, we estimate the origin of Schizophora within the Cretaceous-Paleogene (K-Pg) boundary, about 68.3 Ma. Detailed analyses of the blowfly family (Calliphoridae) place its origin at 22 Ma, concomitant with the radiation of grazing mammals. The emergence of ectoparasitism within calliphorids was dated 6.95 Ma for the screwworm fly and 2.3 Ma for the Australian sheep blowfly. Varying population histories were observed for the blowfly *Chrysomya megacephala* and the housefly *Musca domestica* samples in our dataset. Whereas blowflies (n = 50) appear to have undergone selective sweeps and/or severe bottlenecks in the New World, houseflies (n = 14) display variation among populations from different zoogeographical zones and low levels of gene flow. The reported high-throughput mitogenomics approach for insects enables new insights into schizophoran diversity and population history of flies.

True flies are insects that undergo complete metamorphosis and belong to the two-winged insects order Diptera. They represent one of the most diverse groups in the Kingdom Animalia^1^ and are structured into two major sub-orders: Lower Diptera (mosquitoes) and Brachycera (short-horned flies). Worldwide, more than 150,000 species have been described^2^, making them one of the most successful groups on Earth that occupy almost every terrestrial niche. They parasitize plants and animals, act as biological and mechanical vectors of diseases, serve as biological control agents, as well as model organisms for science^3^. The clade Schizophora contains the majority of the family level diversity^3^,^4^ among Dipterans and represents a recent rapid radiation of lineages. The resulting relationships among Schizophora families remain a challenge for fly phylogeny^2^,^3^. The rapid radiation in combination with a low extinction rate has led to a diversity that surpasses even the number of all terrestrial species of vertebrates^2^,^3^,^4^. The scarcity of fossil records and the sparse availability of genetic data make Schizophora an interesting target for large-scale molecular evolutionary analyses. These analyses were historically based on mitochondrial markers consisting of single genes or short sequence intervals. More recently, complete mitochondrial genomes have become commonly used for in-depth phylogenetic and population studies^5^. In particular for vertebrates, generation of complete mitochondrial genomes is well established^6^, including those of extinct species^7^,^8^,^9^,^10^. Sequencing of complete mitochondrial genomes has been proven to refine resolution of relationships both between and within species^11^,^12^,^13^,^14^,^15^,^16^, in addition to improving the characterization of genome evolution^17^,^18^ and patterns of substitution rate^19^.

In contrast, the sequencing and assembly of complete mitochondrial genomes of invertebrate species has progressed at a much slower pace. This is especially true for insects, whose mitochondrial genomes have been obtained by isolation from whole cell lysates in combination with restriction fragment or shotgun sequencing. Those initial datasets served as references for the further development of universal primers^17^,^20^,^21^,^22^,^23^. Interestingly, even a decade after the emergence of next-generation sequencing (NGS), most invertebrate mitochondrial genomes are still based on primer walking and Sanger sequencing^16^,^24^,^25^,^26^, resulting in an underrepresentation of molecular studies on invertebrate evolution, compared to other taxa.

An alternative approach uses large amplicons (several kilobases in length) generated by long-range PCR in a shotgun approach to assemble these mitogenomes. A number of arthropods mitochondrial genomes have been successfully amplified by this method using sets of conserved primers^27^,^28^,^29^ and sequenced on current versions of NGS platforms. Most of the generated data has been focused on the description of a species’ mtDNA sequence^30^,^31^,^32^,^33^,^34^,^35^, and in a few instances on population studies^29^,^36^. This approach is limited to closely related species, as larger evolutionary distances often disrupt the annealing sites of the universal primers.

Studies targeting a wide range of insect diversity therefore depend on more universally applicable methods. One such approach is the whole genome shotgun (WGS) sequencing of total DNA. This approach can be applied to whole animals or specific body parts, allowing for morphological preservation and molecular analysis of the same specimen. In addition, due to the short DNA read length of NGS data, partially degraded samples may be analyzed successfully.

In order to assess the applicability of the above approaches, we generated a total of 91 complete mitochondrial genomes for 32 different species of flies from the Schizophora radiation. Mitochondrial genomes were sequenced from whole insects, body parts and ethanol-preserved specimens using three different techniques: (i) a combination of long-range PCR and shotgun sequencing; (ii) WGS sequencing of genomic DNA using Illumina short reads; and (iii) WGS sequencing of genomic DNA using long reads from single molecule real time (SMRT) sequencing. These strategies allowed us to increase the taxonomic breadth of dipteran phylogenies and calculate divergence dates for major Schizophora clades. In addition, we inferred the population structure of two important mechanical vectors of diseases in different continents, the Oriental latrine blowfly *Chrysomya megacephala* and the housefly *Musca domestica*. We show that genetic information of a wide-variety of species can be recovered, even if only small body parts or degraded DNA are available. Our results contribute to the comparative mitogenomics and population-based analyses of invertebrates and enable the generation of molecular data in an automated high-throughput fashion for insects in general.

## Results

### mtDNA sequencing strategies and coverage

**Figure 1:**
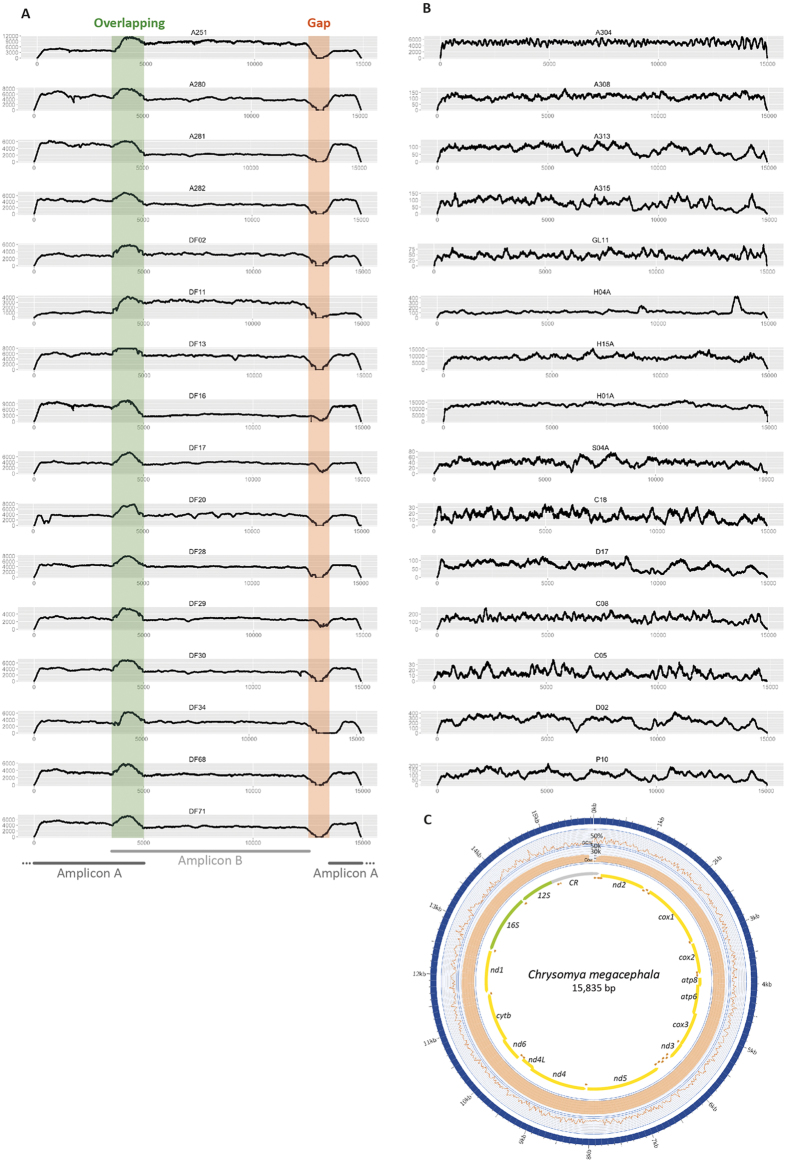
Coverage plots. (**A**) Coverage of mitogenomes from 16 species of Schizophora generated by long-range PCR and shotgun sequencing. Highlighted regions indicate an overlap (green) and the 16S gap (orange) region between the two amplicons. Gaps in 16S sequence were further closed through standard PCR and Sanger sequencing. (**B**) 16 mitogenomes assembled from short reads generated by whole genome sequencing. Both strategies used short reads from MiSeq or HiSeq Illumina platforms to generate high-quality assemblies. (**C**) Coverage plot of the complete mtDNA assembled with long reads generated with SMRT sequencing technology. The scheme shows the complete mtDNA of *C. megacephala* (sample F03) assembled with 15,835 bp. First track shows the low GC content (23.5%). Orange bars on the second track refer to the coverage. The innermost track shows gene order in each mtDNA strand. Yellow arrows denote PCGs, green arrows show rRNA subunits and orange arrows refer to tRNAs.

For the 32 schizophoran species sequenced, 16 mtDNAs were assembled using a combination of long-range PCR and shotgun sequencing, and 16 were recovered using the WGS technique. The number of reads generated and mapped against the mtDNA reference is in Supplementary Table S1. For amplicon sequencing, the mtDNA coverage ranged from 2492-fold (*Morellia lopesae*) to 8830-fold (*Chloroprocta idioidea;* Supplementary Table S1). On average, 80.16% (±10.55) of the reads were aligned to the reference. As expected, the strategy using WGS provided a lower percentage of the total reads aligned to the reference (1.43% ± 1.22), since the nuclear genome and the metagenome are also generated. However, mtDNA-assigned reads were sufficient to generate high coverage assemblies, ranging from 12-fold (*Muscina levida*) to 13,169-fold (*Phormia regina*; Supplementary Table S1), dependent on the total number of reads generated for each library. The WGS using SMRT platform yielded a total of 72,445 pre-assembled reads (p-reads) that contain the full mtDNA sequence of the species *C. megacephala*. This strategy generated a mitogenome with 21,488-fold coverage (sample F03, Supplementary Table S2), with 99.94% identity to the reference genome (NC_019633.1^26^). Also, the PacBio SMRT platform was the only technique that allowed for the complete recovery of mtDNA, including the full control region (CR). The mitochondrial coverage of short-read assemblies is shown in Fig. 1A,B for all 32 schizophoran species, and the assembly with long reads is in Fig. 1C. The coverage of remaining samples used for population analyses is in Supplementary Fig. S1 and in Supplementary Table S2.

### Phylogenetic analyses

**Figure 2:**
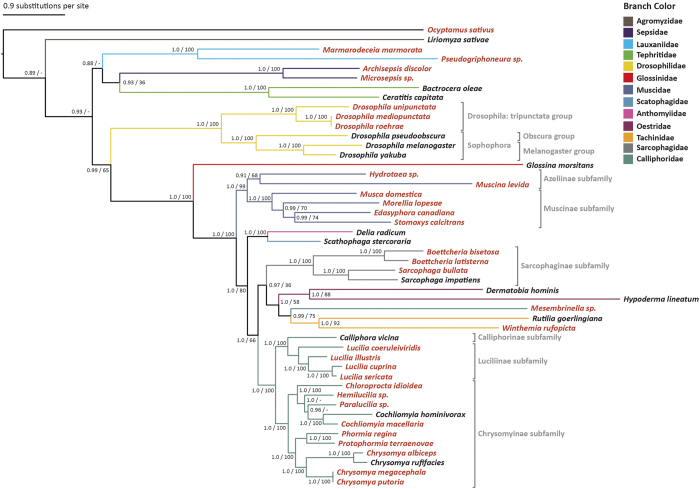
Phylogenetic tree inferred for the Schizophora clade. Phylogenetic trees were generated for the 48 mitochondrial genomes analysed through the favoured partition scheme mtDNA11PF (see Methods and Supplementary Table S4 for details) under BI and ML methods. The ML tree topology is identical to the BI tree (shown) with exception of relationships among some Acalyptratae lineages. Values at branches refer to node supports of BI posterior probabilities and ML bootstrap proportions among 1000 replicates, respectively. Bootstrap supports below 50 were omitted (−). In red we highlight the new mitogenomes sequenced in this work.

A total of 48 mitochondrial genomes, comprising 13 families and 8 superfamilies of Schizophora (see Supplementary Table S3 for full phylogenetic dataset and Fig. S2 for a map with sampling sites) were used for phylogenetic inferences under maximum likelihood (ML) and Bayesian inference (BI) methods. Phylogenetic trees inferred under different methods and partitioning strategies were concordant, with a few exceptions regarding the relationships among some Acalyptratae lineages. Although a major topological difference was not observed among analyses, differences in Bayes factor model comparisons were statistically significant (>200 in likelihood values; Supplementary Table S4), always favoring more complex partitioning strategies. The profiles of intra and inter-familial genetic distances (Supplementary Fig. S3) suggest different evolutionary rates among protein coding genes (PCGs), justifying the use of more complex strategies. The topology inferred using the Bayes Factor-favored strategy (mtDNA11PF) was the most recovered topology in our analyses and is shown in Fig. 2.

Schizophora was recovered as a monophyletic group, containing the two subsections Acalyptratae and Calyptratae. Both subsections were traditionally classified based on the size of the lower calypter as a synapomorphy, which showed to be unreliable. The Acalyptratae is a large subsection that lacks a comprehensive phylogenetic analyses and show contradictory synapormorphies^3^,^37^. The Drosophilidae family (superfamily Ephydroidea) was recovered as the sister-clade of Calyptratae in both BI and ML inferences, although modestly supported by bootstrap values (Posterior Probability [PP] = 0.99 and bootstrap [BS] = 65, Fig. 2). A monophyletic Calyptratae clade (PP = 1.0, BS = 100) was nested inside the paraphyletic Acalyptratae grade.

Calyptratae is one of the most diverse and successful fly groups^37^, classically divided into three superfamilies: Hippoboscoidea, Muscoidea and Oestroidea, all sampled in our analyses. Within Calyptratae, the Hippoboscoidea *Glossina morsitans* was recovered as the sister-taxon of a clade composed of a paraphyletic Muscoidea grade and a monophyletic Oestroidea, with the Muscoidea clade (Anthomyiidae + Scathophagidae) as sister group of Oestroidea (PP = 1.0, BS = 80). These relationships are in accordance with recently published molecular phylogenies based on combined datasets of nuclear and mitochondrial genes^2^,^38^,^39^. The monophyly of Calyptratae is well supported in literature^3^,^4^, but its position within the main Schizophora clade remains controversial. The search for the sister-taxon of Calyptratae shows a close relationship with both the Acalyptratae superfamilies Ephydroidea (including Drosophilidae) and Tephritoidea (including Tephritidae)^2^,^40^, as also observed in this study. A complete split between Calyptratae and Acalyptratae, both being monophyletic, has also been proposed^4^ but little support for this hypothesis has been found.

Oestroidea interfamilial relationships were also consistent among BI and ML analyses, with a monophyletic core-Calliphoridae (*sensu*^38^,^39^,^41^), excluding Mesembrinellinae (PP = 1.0, BS = 100). This group was recovered as sister-group of a clade composed of (Sarcophagidae + (Oestridae + (Tachinidae + *Mesembrinella* sp.))) (PP = 0.99, BS = 66). In literature, Oestroidea interfamilial relationships have been contentious, with few agreements among different studies^4^,^38^,^39^,^41^,^42^,^43^,^44^. The exclusion of *Mesembrinella* sp. from the core-Calliphoridae lineage and its placement as sister group of Tachinidae (PP = 1.0, BS = 75) is particularly interesting, as recurrent studies show support for the creation of a distinct family named Mesembrinellidae within Oestroidea. Our results support this taxonomical revision and the creation of Mesembrinellidae, comprising *Mesembrinella* and other small related genera of the subfamily Mesembrinellinae^39^,^45^.

### Estimation of divergence time

**Figure 3:**
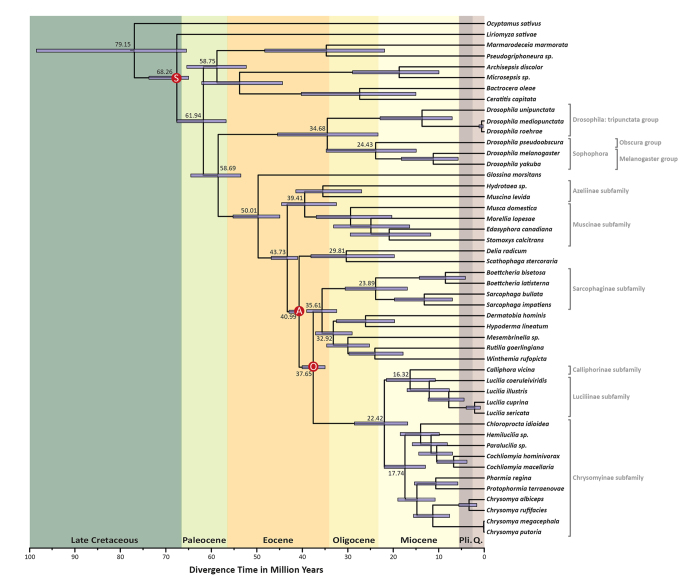
mtDNA time tree of Schizophora. Divergence timescale for the Schizophora clade inferred under Bayesian uncorrelated relaxed clock method from 48 complete mitochondrial genomes. Node values indicate mean estimated divergence times in million years (Ma) and bars indicate 95% credibility intervals. Calibration points for Schizophora crown (S), Anthomyiidae stem (A) and Oestroidea crown (O) fossil constraints are shown in red circles. Pliocene and Quaternary at geological time scale are shown as “Pli.” and “Q.”, respectively.

The Bayesian uncorrelated relaxed clock^46^ was used to estimate the divergence timescale of the Schizophora. Fossil constraints related to the radiation of Schizophora (S), Oestroidea (O) and the origin of Anthomyiidae (A) were included for molecular clock calibration. Our results (Fig. 3) place the origin of the Schizophora clade within the Late Cretaceous to Paleogene periods (known as K-Pg boundary), about 68.3 Ma (95% credibility interval [CI]: 65.73–70.86 Ma).

Schizophora is usually split into two major groups, namely Acalyptratae and Calyptratae. Controversial relationships within the Acalyptratae clade are often reported because of its extreme diversity. The divergence estimation obtained for this group shows that all sampled families have their last common ancestor in the early Paleogene, around 62.0 Ma. This timescale is corroborated by the earliest Agromyzidae specimen found in Baltic amber deposits dated to 64 Ma^47^,^48^. Within the family Drosophilidae, the split of *obscura* and *melanogaster* groups was dated back to the Oligocene (~24.4 Ma, CI: 14.94–34.76 Ma), after the divergence of *Drosophila* and *Sophophora* subgenus in the late Eocene (~34.7 Ma, CI: 23.34–45.43 Ma). The divergence times inferred for the family Drosophilidae are similar to those based on the complete genomes of 12 *Drosophila* species, which estimated that *Drosophila* and *Sophophora* split 40 Ma and *obscura* and *melanogaster* groups were split about 26 Ma^49^.

For the Calyptratae subsection, the most recent common ancestor was inferred to exist in the early Eocene, about 50 Ma (95% CI: 44.95–55.22 Ma). Within Muscoidea, the divergence of Muscidae and Anthomyiidae families was dated to 43.7 Ma (95% CI: 41.0–46.8 Ma). Main radiation of Muscidae was estimated to have occurred about 39.41 Ma (95% CI: 32.5–44.54 Ma), followed by the emergence of the Muscinae subfamily in the late Eocene to early Miocene, about 29.48 Ma (95% CI: 20.3–36.9 Ma). These inferences support recent findings, suggesting that Muscidae radiation is more recent than previous estimations and probably took place near the final stages of the Gondwana breakup^50^.

The Oestroidea radiation is nested in a paraphyletic Muscoidea grade and was estimated to have diverged in the late Eocene, about 37.65 Ma (95% CI: 35–40 Ma). Those estimations are in agreement with Wiegmann *et al.*^2^, who used nuclear and mitochondrial genes from eleven Oestroidea species. However, our estimations and tree topology differ from Zhao *et al.*^51^, who used complete mitochondrial genomes from eight Oestroidea species and could not recover their monophyly in ML analyses. The present study used a broader diversity of twenty-five Oestroidea species, including its main families. In particular, the inclusion of a Mesembrinellinae sample was crucial to shed light on controversial phylogenetic relationships within the Oestroidea superfamily, which has been the subject of taxonomical debate^38^,^39^,^52^. Our results support the placement of Mesembrinellinae in a different family, distinct from the core-Calliphoridae (comprised of the subfamilies Calliphorinae, Chrysomyinae, Lucillinae, Toxotarsinae and Melanomyinae^41^), as proposed by Marinho *et al.*^39^ and Guimarães^45^. Moreover, our results suggest that the diversification of Calliphoridae took place 10–15 million years after the divergence of *Mesembrinella sp.* (Fig. 3).

The family Calliphoridae (*sensu lato*) encompasses ~1500 species commonly known as blowflies. The larvae of most calliforids have saprophagous habits, thus playing an important role in recycling organic matter. This family also includes specific parasites of mammals and birds that cause larval infestations known as myiasis^53^. Obligate parasites feed on live tissues of cattle and warm blooded mammals, causing losses to the agropecuary sector^54^. The Calliphoridae family is a particular challenge for Oestroidea relationships, since its composition and monophyletic status has been controversial. Our analyses place the last common ancestor of the core-Calliphoridae at the geological border of Oligocene and Miocene (~22.4 Ma), followed by the rapid radiation of the subfamily Chrysomyinae (~17.74 Ma) and Calliphorinae + Luciliinae sister-lineages (~16.32 Ma). These results are similar to those estimated for the carrion-breeding blowflies from Australia^55^.

### Population analysis

We evaluated the population diversity and structure of two species in urban centers on different continents: the Oriental latrine blowfly *Chrysomya megacephala* (Fabricius, 1974) and the housefly *Musca domestica* (Linnaeus, 1758). Both species are among the most abundant and important insects around the world and are widely distributed^56^. Because of their mobility and association with human habits, they can act as mechanical vectors of diseases by transporting microorganisms from feces, garbage and carcasses to humans and animals^53^. The population analyses of blowflies and houseflies were based on samples collected for whole genome metagenomic studies in urban and rural areas of South and North America, Australia and Singapore (see Supplementary Table S5 and Fig. S2 for location and map). The increasing number of metagenomic studies in insects generates the full microbiome of individuals, but mitogenomes also can be successfully assembled at deep coverage (Supplementary Table S2). In this work, metagenomic datasets generated for 64 specimens of blowflies and houseflies were used as a model to advance mitogenomics of insects at population level.

**Figure 4:**
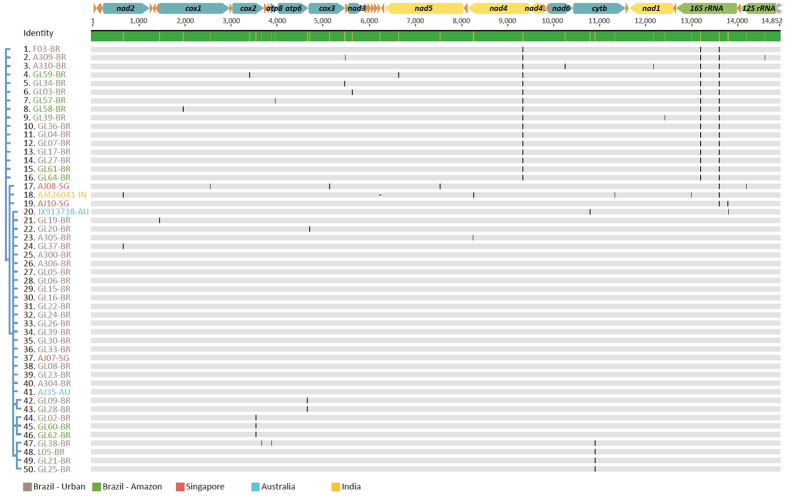
Blowflies’ mitochondrial variation. Alignment of complete mtDNA sequences of 50 specimens of the Oriental latrine blowfly *C. megacephala* (control region, shown in grey in the top linear mtDNA scheme, is excluded). Substitutions are shown as a black bar along the mtDNA for each sample. Consensus tree on the left was inferred through MrBayes. Mitochondrial PCGs coded in the major strand are in blue and those coded in the minor strand are in yellow. rRNA subunits are shown in green and tRNAs in orange. The direction of the arrows indicates the strand location (“+strand” to the right and “− strand” to the left).

The analyses of 50 mitogenomes of *C. megacephala* revealed a low genetic diversity (Fig. 4), with a total of 22 haplotypes and only 34 variable sites (1 indel in non-coding region) along 14,852 sites analyzed. The mean number of pairwise differences over all sequences was 2.87 ± 2.0, while the average nucleotide diversity (p-distance) was 0.00019 ± 0.0001. Samples from Brazil (BR; n = 44), Singapore (SG; n = 3), Australia (AU; n = 2) and India (IN; n = 1) were grouped and the p-distance within groups was 0.00027 for SG, 0.00018 for BR and p = 0.00013 for AU. The average p-distance between groups (p = 0.00031 ± 0.0001) was larger than within groups, but overlapping values between maximum intra-group and minimum inter-group distance is noticeable. The average number of pairwise differences between and within populations, as well as Nei’s distance (d) is shown on Supplementary Fig. S4. The fixation index (F_ST_) is low among the Australian, Singaporean and Brazilian sub-groups ranging from 0.03 to 0.15, indicating admixture among these populations. The Indian sub-group showed isolation from others, but was removed from the pairwise F_ST_ analyses due to low sampling.

**Figure 5:**
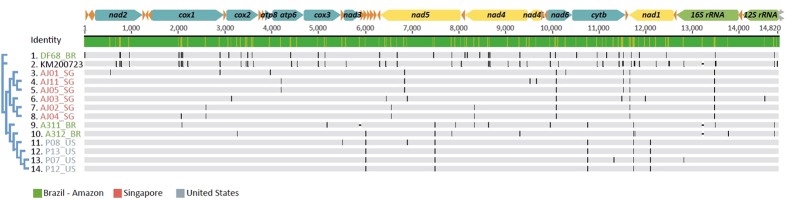
Houseflies’ mitochondrial variation. Alignment of complete mtDNA sequences of 14 specimens of the housefly *M. domestica* (control region, shown in grey in the top linear mtDNA scheme, is excluded). Substitutions are shown as a black bar along the mtDNA for each sample. Consensus tree on the left was inferred through MrBayes. Mitochondrial PCGs coded in the major strand are in blue and those coded in the minor strand are in yellow. rRNA subunits are shown in green and tRNAs in orange. The direction of the arrows indicates the strand orientation (“+strand” to the right and “− strand” to the left).

The population analyses of 14 samples of *M. domestica* (Fig. 5) show a total of 13 haplotypes with 100 variable sites (3 indels in non-coding regions) out of 14,820 sites. The overall mean number of pairwise differences was 21.65 ± 17.4 and the average p-distance was 0.0015 ± 0.0012. Houseflies from Brazil (BR; n = 3), United States (US; n = 4) and Singapore (SG; n = 6) were grouped in populations, while the reference mitogenome KM200723^57^ could not be assigned to any location due to lack of this information in GenBank metadata. The p-distance within groups was 0.0022 for BR, 0.00013 for US and 0.0005 for SG. The average p-distance between groups was p = 0.0014 ± 0.0004. The overlapping values within and between populations are due to the sample DF68 from Brazil, which does not cluster with other Brazilian samples. The average number of pairwise differences between and within populations and the Nei’s distance (d) are shown on Supplementary Fig. S5. Together, these results suggest that the Brazilian population is the most diverse. The F_ST_ matrix in Supplementary Fig. S5 shows significant values of F_ST_ between the SG group compared to the others, which causes the Asian and American groups (except for DF68) to cluster separately. The F_ST_ matrix indicates intermediate to high levels of isolation of *M. domestica*, forming two major clusters (US + BR groups and SG group).

## Discussion

The three strategies used to obtain the complete mitogenomes of a highly diverse group of species (~80 million years of evolution) successfully yield mtDNA sequences from small parts, dried specimens and fresh samples. Although providing high coverage of the mtDNA, the PCR + WGS technique prevents the sequencing of the CR and shows a variable coverage pattern (Fig. 1), depending on the overlap of amplicons. However, this method produced sequences from small parts (wing, muscles and legs) and tiny specimens, proving to be a reliable strategy for mtDNA sequencing. The WGS technique provided a uniform depth of coverage, either with short or long reads (Fig. 1B,C). The advantage of using the short reads is the large number of sequences generated in a highly multiplexed run that currently allows for up to 96 indexed samples. Despite the large amount of DNA required as input, the SMRT platform yields do not allow for more than 10 multiplexed samples, otherwise the depth of coverage is low. However, the long-read assembly was able to generate a full-length mitogenome that included the complex CR sequence. The CR of dipterans is highly biased towards A + T (>90% content) and forms secondary structures that usually hinder its sequencing^58^.

In a comparative perspective, all techniques can be fully automated with liquid handling systems and analyzed with a high-throughput pipeline. The price range to generate mitogenomes with different strategies and sequencing platforms is in Supplementary Table S6. The WGS using short reads is the cheapest approach to generate nearly complete mitogenomes (USD 103), followed by the combination of long-range PCR and shotgun sequencing (USD107). The use of the SMRT platform was capable of generating full-length mtDNA, but was demonstrated to be the most expensive (USD 358). Therefore, the choice of the mitogenomic approach will largely rely on the needs for complete or near complete genomes and the resources available.

The phylogenetic and molecular dating analyses showed that the main radiation of Schizophora took place in the Paleogene period, particularly in the Paleocene and Eocene epochs, when most schizophoran families diverged. Some authors argue that this clade is more diverse today in the tropics than in other geographic zones because of climate change during the early Eocene, leading to higher temperatures^59^. The Paleogene period witnessed series of global changes during a cooling period with intervals of global warming that reached its peak in the Paleocene-Eocene Thermal Maximum^60^. The diversification of insects, in particular phytophagous and mycophagous acalyptrates, also seems to be correlated with the radiation of angiosperms^59^. Flowering plants dominated environments in the Late Cretaceous^61^ and are associated with the radiation of pollinators and herbivorous insects during the Paleogene period. The calyptrate diversity was indirectly affected by the angiosperms radiation, as is the case for parasites of phytophagous insects, such as flies from the family Tachinidae. Moreover, low extinction rates also were fundamental for the diversification and survival of Schizophora clade during the K-Pg mass extinction^2^.

Papavero^62^ hypothesized that the emergence of early mammals in the Cretaceous also affected the diversification and radiation of the Oestroidea superfamily (Calyptratae), since many lineages present a parasitic relationship with vertebrate hosts^63^, mainly mammals. An example is the Oestridae family that parasitizes mammals, usually exhibiting a host-specific endoparasitism. Oestrid parasites may have originated from rodent parasitism^64^ during the K-Pg boundary, but the major diversification of Oestridae began with the wide radiation of mammals during the Paleogene.

The best sampled group in our analyses comprises the monophyletic core of the family Calliphoridae, *sensu* Rognes 1997. This family contains species known as blowflies and screwworm flies that can impact human and animal health. The branch lengths and diversification of species within Calliphoridae (Figs 2 and 3) suggest that most of its diversity arose in the last 20 million years. The rise and diversification of the core-Calliphoridae in the early Miocene may be related to peculiar geological changes and evolutionary processes that created new niches to be occupied and, therefore, triggered an adaptive radiation of blowflies. The Oligocene-Miocene border was an important period for the diversification and spread of large mammals, after global warming was followed by a drought. The dry conditions led to the replacement of tropical forests with grasslands^65^,^66^ that are associated with the radiation of grazing animals. Herbivores became more common starting in the Oligocene, increasing the diversification of carnivores. In particular, artiodactyl species flourished in this grassy landscape and their dispersion became facilitated with geological changes that closed the connection of the Mediterranean Sea and Indian Ocean^67^, linking Africa and Eurasia. Furthermore, East Asia and North America were connected through the Bering land bridge^67^, allowing the dispersal of animals and plants to the New World. These conditions also may have created the perfect landscape for calliforid diversification, with an increase in organic matter widely produced by large mammals. To corroborate this hypothesis, we analyzed the pattern of diversification of the family Sarcophagidae (flesh flies), which has feeding and breeding habits similar to Calliphoridae, and lays their eggs in decaying organic matter. The flesh flies show a similar pattern of diversification in our analyses with a common ancestor being placed ~23.9 Ma (CI: 16.9 – 30.57 Ma). In-depth analyses of published trees of Oestroidea flies^38^,^39^,^68^,^69^ revealed that branch lengths and genus diversification are similar between blowflies and flesh flies. Taken together, geological and biological conditions could have increased the availability of manure and organic matter that served as a trigger for the radiation of blowflies and flesh flies during the Miocene epoch. After genus diversification about 15 Ma, flies may have adapted to specific hosts in the family Calliphoridae, ultimately leading to the evolution of ectoparasitic lifestyles dated at 6.95 Ma (CI: 3.77–10.41 Ma) for the obligatory parasite *C. hominivorax* (screwworm fly) and 2.28 Ma (CI: 0.86–3.96 Ma) for the facultative parasite *L. cuprina* (Australian sheep blowfly). The ectoparasitic habit within the family Calliphoridae could have emerged in parallel, after continents drifted. This could explain the non-overlapping distribution of parasitic flies species in different continents.

The mitogenomic population analysis indicated low population subdivision (F_ST_ < 0.15) and genetic distance among *C. megacephala* sampled in Australia, Singapore and Brazil (Fig. 4 and Supplementary Fig. S4), suggesting a panmitic population. The average number of differences between all individuals analyzed is 2.9 (4.6 between populations), which is equivalent to the genetic diversity found in extinct animals such as the Tasmanian tiger^70^. *C. megacephala* invaded Southern Brazil in the mid-70’s and rapidly spread across South America^71^. Initially, the introduction was reported to be from Africa^72^, but a different hypothesis considered the Australasian region as the source of introduction^73^. Our results show that haplotypes from Singapore and Australia are found in Brazilian samples, thus suggesting that the introduction may originate from the Australasian region. However, the source of the introduction in South America is uncertain and analysis would be improved with samples from ancestral populations from Africa. The Oriental latrine fly can occupy a variety of niches, and is found in diverse habitats ranging from savannas and rainforests to urban centers around the world. A previous study suggested that the synanthropic form of *C. megacephala* has its origin in New Guinea^74^. However, analyzing the variation among samples from urban areas in Sao Paulo and in the rainforest in Amazon, we could not find exclusive haplotypes in urban and natural environments. Likely, the low mtDNA diversity found in this species is caused by a severe bottleneck during introduction into the New World and subsequent spread of one or a few lineages of mtDNA carried by a few females. Alternatively, potential linkage of mtDNA and insect symbionts was also considered as a cause of the low mtDNA variation^75^. The *Wolbachia* symbiont is widely spread in insects^76^ and was found in all samples of *C. megacephala* analyzed in this work. *Wolbachia* can be vertically transmitted by females and affects the sex ratio of offspring through feminization, parthenogenesis, male killing and cytoplasmic incompatibility in invertebrates^76^. The extent of impact caused by *Wolbachia* in the genetic variation and structure of *C. megacephala* is under investigation and early results show a low genetic diversity in *Wolbachia* infecting different populations (data not shown). Additionally, it was previously described that infected blowflies of the species *Protocalliphora sialia* (Calliphoridae) carry less mtDNA diversity than uninfected blowflies, suggesting a selective sweep and linkage disequilibrium that may drive geographical and genetic structure^77^. If the same pattern occurs in *C. megacephala*, mtDNA haplotypes correlated to the initial infection of the symbiont could hitchhike, resulting in reduced mitochondrial diversity due to indirect selection rather than a bottleneck.

The housefly is a widespread species that is found on all continents and survives in temperate and tropical climates. It is reported that populations from temperate climates undergo multiple bottlenecks because of low temperatures that reduce the population size drastically^78^. Such bottlenecks might be the cause of the lower diversity found in the US populations than in populations in tropical regions (Brazil and Singapore), where breeding can take place year-round. The presence of different haplotypes in the New World (Neartic and Neotropical zones) is consistent with the hypothesis that *M. domestica* underwent multiple introductions from Paleartic regions, where the species likely originated^79^. Also consistent with this hypothesis, we find clustering of haplotypes into two groups, one from the Americas (US + BR samples) and another encompassing the Indomalayan zoogeographical zone, represented by SG samples. Despite the observation of clusters from different regions, *M. domestica* shows a low mitochondrial genetic diversity, with an average of 21.6 nucleotide differences between two individuals, but a significant population differentiation. This indicates that most genetic variation is among populations of different zoogeographical zones and that the gene flow among them is low. Genetic drift might be an explanation for this pattern, but differential sex-ratio also may influence female fitness. This could lead to the increase of eggs laid by one female genotype and enhance survivorship of the offspring^80^, affecting the fixation of mitochondrial lineages in different locations. Further investigations are necessary to correlate mitochondrial haplotypes with nuclear genotypes and to address ancestral populations.

Population approaches such as those shown in this work highlights the potential of mitogenomics developed from environmental sequencing projects. The evaluation of the genetic diversity in a population level proved to be reliable, at relatively low costs, from a complex mixture of reads initially generated for a metagenomic study. The development of new high-throughput pipelines to efficiently analyse molecular data from invertebrates also provides insights into species relationship, population structure and evolution of one of the most diverse groups of animals on Earth. Given the impact of flies on environmental and human health, the availability of new molecular data generated in this work will provide a phylogenetic timeframe reference for Schizophora, with estimates based on full mitogenomes and fossil records that can aid future comparative studies in Diptera.

## Methods

### Sampling, DNA extraction and mtDNA sequencing

Specimens of the suborder Brachycera were collected with an entomological net by sweeping or with decomposing fish as a bait to attract adults. Samples were collected in Sao Paulo and Amazonas states in Brazil, in Pennsylvania in the United States, Western Australia in Australia, and in Singapore. Representative sequences for 32 species were used for phylogenetic analysis (Supplementary Tables S1 and S3), while multiple sequences of *C. megacephala* and *M. domestica* were used in population-level analyses (Supplementary Table S2 and S5) using datasets generated for a metagenomic study. Specimens were identified through morphological traits and with the *cox1* gene as a DNA barcode^81^.

For most of the samples, two main strategies were adopted to generate mtDNA sequences from short reads: (i) coupling long-range PCR with sequencing on a MiSeq (Illumina Inc.) platform, and (ii) WGS sequencing on a MiSeq or HiSeq2000 (Illumina Inc.). A third strategy was also performed using single molecule long reads generated by a PacBio RSII (Pacific Biosciences). Total genomic DNA was extracted from legs or thoracic muscle of dried and ethanol-preserved specimens using an adapted protocol for DNAzol**^®^** (Invitrogen)^82^ or with Spin Tissue Mini-Kit (Invitek), following manufacturer’s instructions. Frozen specimens had DNA extracted with Phenol/chlorophorm^83^ or using the DNeasy Blood and Tissue Kit (Qiagen), following the manufacturer’s instructions.

Long-range PCR was performed to generate two amplicons of 8 and 9.2 kb. Reactions were carried out following a protocol with optimized primers described previously^28^. Amplicons were purified with illustra GFX™ Purification Kit (GE Healthcare) and quantified by Qubit^®^ (Life Technologies). Equal amounts of the two amplicons were pooled to a final amount of 1 μg for each sample. For the WGS approach, DNA was quantified by Qubit^®^ and analyzed on an Agilent 2100 Bioanalyzer with the DNA 12000 kit (Agilent Technologies). A total of 1 μg of total DNA was used for sequencing.

Pooled amplicons and genomic DNA were sheared to 300 bp using a Covaris S220 focused-ultrasonicator (Covaris Inc.), according to Illumina’s protocol. Library construction was fully automated using SPRIworks Fragment Library System I (Beckman Coulter) with TruSeq Indexed Adapters (Illumina Inc.). Libraries were size-selected with Pippin Prep (Sage Science) and enriched according to the TruSeq kit protocol. Each library was quantified using Quant-iT™ PicoGreen**^®^** (Invitrogen) and qPCR was performed according to KAPA SYBR**^®^** FAST qPCR kit instructions (Kapa Biosystems). Equimolar amounts (1 nM) of 24 indexed libraries were pooled for multiplex sequencing. A total of 8 pmol of each pool was added to a flowcell and sequenced on a MiSeq or HiSeq 2500 (Illumina Inc.) platform using 150 × 150 bp paired-end run.

For SMRTbell™ library construction, high molecular weight DNA was quantified by Qubit**^®^** and the OD_260_/OD_280_ ratio was assessed by a Nanodrop (Thermo Scientific). A total of 10 μg of DNA (OD_260_/OD_280_ = 1.8) was pooled from siblings of the same offspring (same mtDNA lineage) and sheared to 9 and 15 kb using a Covaris S220. SMRTbell™ libraries’ yield and size distribution were measured on an Agilent 2100 Bioanalyzer using the High Sensitivity Kit chip. The libraries were fully constructed and sequenced on a PacBio RSII at the Pacific Biosciences headquarter (Menlo Park, USA).

### Mitochondrial Genome Assembly and Annotation

The representative mtDNA sequences for each species were generated using a reference-assisted assembly approach. The short Illumina sequences were aligned to the mtDNA of *Exorista sorbillans* (GenBank accession NC_014704.1) using LASTZ^84^, requiring an identity of 75% or greater over at least 90% of the sequence. The sequences that aligned uniquely to the reference were input to YASRA^85^ to generate a consensus sequence. The resulting contigs were then refined by realigning all the short reads to them using a BWA/SAMtools^86^,^87^ pipeline with default parameters. The assembled representative sequences of *C. megacephala* and *M. domestica* were used as references for individuals of the same species. These alignments were generated using BWA with default parameters, and the consensus sequences were generated using SAMtools.

The blowfly genome was assembled with Falcon v 0.2.2 and was polished with Quiver in SMRTanalysis portal v 2.3. The parameters are available upon request. The mitochondrion contig was extracted from the rest of the blowfly contigs based on its similarity with the reference NC_019633.1.

For each of the 32 species, annotation of PCGs was assisted by DOGMA^88^. Transfer RNAs (tRNA) were annotated based on tRNAscan-SE 1.21^89^,^90^ predictions and ribosomal RNAs (rRNA) on sequence and positional homology. For the population analyses, mtDNA sequences of the same species were aligned with MUSCLE^91^ and the annotation was automated using Geneious^®^ 8.1.3. Annotated files were generated using Sequin 13.70 and submitted to NCBI.

### Phylogenetic Analyses

The database MetAmigA^92^ was used to generate the complete mtDNA dataset used for further analyses (Supplementary Table S3). PCGs were individually aligned using MUSCLE^91^ in TranslatorX^93^ server. Alignments were back translated to nucleotide sequences and poorly aligned regions were filtered using Gblocks v. 0.91^94^. Alignments of the 12 S and 16 S rRNAs were conducted in ClustalX v.2.1^95^ and secondary structures were modeled by homology, based on the structures described for *D. melanogaster*^96^, *D. virilis*^96^ and other insects^97^. The variable helix H2077 was predicted *in silico* using mfold v3.0^98^ with default parameters. Secondary structure information was used to guide adjustments in final alignments and is shown in Supplementary Fig. S6.

Evolutionary divergences among mitochondrial genes were accessed indirectly based on the profile of intra- and inter-group (families) genetic distances, calculated using MEGA 6.06^99^ and analyzed with R^100^. Model selection for each gene (considering dsRNA, ssRNA and codon positions) were performed with Mr. AIC 1.4.4^101^. Different *ad hoc* partitioning strategies (by genome, gene or codon) were evaluated for phylogenetic inferences. Optimal combinations of partitions, as determined by PartitionFinder 1.1.1^102^, were also used in phylogenetic inferences. For the BI, partitioning strategies were further improved with the rRNAs secondary structure consensus generated by PHASE 2.0 package^103^. All partition schemes used and the best-fit model for each mitochondrial gene are shown in the Supplementary Table S7.

ML analyses were conducted using GARLI v. 2.01^104^ with two independent search replicates for 25 million generations and 50 individuals per generation. Node supports were evaluated with 1,000 bootstrap (BS) resampling. All phylogenetic analyses were performed on the CIPRES gateway^105^ using the species *Ocyptamus sativus* (Aschiza: Syrphoidea) as the outgroup. Bayesian analyses were carried out using MrBayes v3.2.2^106^. Two independent analyses were run for 50 million generations (sample frequency = 1.000), burn-in set to 25% after checking for convergence (standard deviation of split frequencies < 0.01) and effective sample size ESS ≥ 200. Remaining samples were used to generate a 50% majority-rule consensus tree and node supports were analyzed based on posterior probabilities (PP).

In order to evaluate whether the effects of different partitioning strategies were significant, the resultant BI phylogenies were compared using the Bayes Factor statistic^107^ (Supplementary Table S4), considering the harmonic mean of the likelihood values sampled during the stationary phase of the Markov Chain Monte Carlo (MCMC) run as an estimator for the marginal-likelihoods of the models^101^.

### Estimation of divergence times

There is a growing evidence that substitution rate patterns of Metazoan mtDNA are time-dependent biased^108^. In order to overcome error estimations in recent evolutionary events of Schizophora, we used fossil constraints close to the divergence events of interest and applied a relaxed clock model that allows for substitution rates to vary among lineages, as previously proposed^109^. Divergence times were estimated using the Bayesian uncorrelated relaxed clock method^46^ implemented in BEAST v.2.1.3^110^. Rate variations among lineages were drawn from a log-normal distribution across branches. The Yule birth-death process was used for the tree prior. The favored BI tree was used as starting topology (not fixed) with the mtDNA11PF partition strategy. No significant changes were observed when using the favored ML tree as a starting topology. Three fossil constraints were included for calibration, comprising the radiations of the Schizophora and Oestroidea lineages and the origin of Anthomyiidae (Supplementary Methods for detailed information). A MCMC sampling size of 60 million was used with samples drawn every 1000 steps. Burn-in was set to 25% after checking for convergence and ESS ≥ 200. The maximum clade credibility tree was annotated using TreeAnnotator v.2.1.2. Three independent analyses excluding each of the fossil constraints were performed in order to evaluate their changing effects in estimation of Schizophora radiation. No significant changes were observed in this analysis.

### Population Analyses

Complete mtDNA sequences for multiple samples of *C. megacephala* and *M. domestica* were aligned with mitogenomes available on GenBank^26^,^57^,^111^ using MUSCLE^91^. For *C. megacephala*, the analyses encompassed 50 mtDNA sequences including 14,929 sites. For *M. domestica*, 14,820 sites were considered within 14 mitogenomes. Samples were obtained from a WGS metagenomic dataset and used as models to develop the mitogenomic pipeline. Alignment gaps and the complete control region were removed. The nucleotide substitution model was selected based on jModelTest 2^112^, using the Bayesian information criterion (BIC)^113^. The variable sites were called using Geneious^®^ 8.0.5. ML phylogenetic trees were generated with PHYML v.3.1^114^ using 1000 non-parametric bootstrap replicates under the HKY substitution model. Bayesian analyses were performed with MrBayes 3^106^ using the HKY substitution model with 4 heated chains during 2,000,000 generations, sub-sampling trees every 500 cycles and burn-in of 500,000. Remaining tree samples were used to generate a 50% majority-rule consensus tree.

Estimation of differences over all sequence pairs and p-distance were obtained with MEGA6^99^. The haplotype diversity was calculated using DnaSP v5^115^. The inter-haplotype distance matrix for each population group and the F_ST_ matrix were inferred using Arlequin 3.5^116^.

## Additional Information

**How to cite this article**: Junqueira, A. C. M. *et al.* Large-scale mitogenomics enables insights into Schizophora (Diptera) radiation and population diversity. *Sci. Rep.* **6**, 21762; doi: 10.1038/srep21762 (2016).

## Supplementary Material

Supplementary Information
