# Supplementary material for: Large-scale mitogenomics enables insights into Schizophora (Diptera) radiation and population diversity
Source: Sci Rep. 2016 Feb 25;6:21762. doi: 10.1038/srep21762 (PMC4766414; doi:10.1038/srep21762)
Supplement: Supplementary Information [file srep21762-s1.pdf]

Supplementary Information

Large-scale mitogenomics enables insights into Schizophora (Diptera) radiation and population diversity.

Ana Carolina M. Junqueira[1*], Ana Maria L. Azeredo-Espin[2], Daniel F. Paulo[2], Marco Antonio T. Marinho[3], Lynn P. Tomsho[4], Daniela I. Moses[1], Rikky W. Purbojati[1], Aakrosh Ratan[5], Stephan C. Schuster[1]

[1] Singapore Centre on Environmental Life Sciences Engineering, Nanyang Technological University, Singapore, 637551.

[2] Centro de Biologia Molecular e Engenharia Genética and Departamento de Genética, Evolução e Bioagentes, Universidade Estadual de Campinas, Campinas, SP, Brazil, 1303-875.

[3] Faculdade de Filosofia Ciências e Letras de Ribeirão Preto, Departamento de Biologia, Universidade de São Paulo, Ribeirão Preto, SP, Brazil, 14040-901.

[4] Center for Comparative Genomics and Bioinformatics, Department of Biochemistry and Molecular Biology, The Pennsylvania State University, University Park, PA, USA, 16802.

[5] Department of Public Health Sciences and Center for Public Health Genomics, University of Virginia, Charlottesville, Virginia 22908, USA

[*] Corresponding author: anacmj@gmail.com; anacarolina@ntu.edu.sg

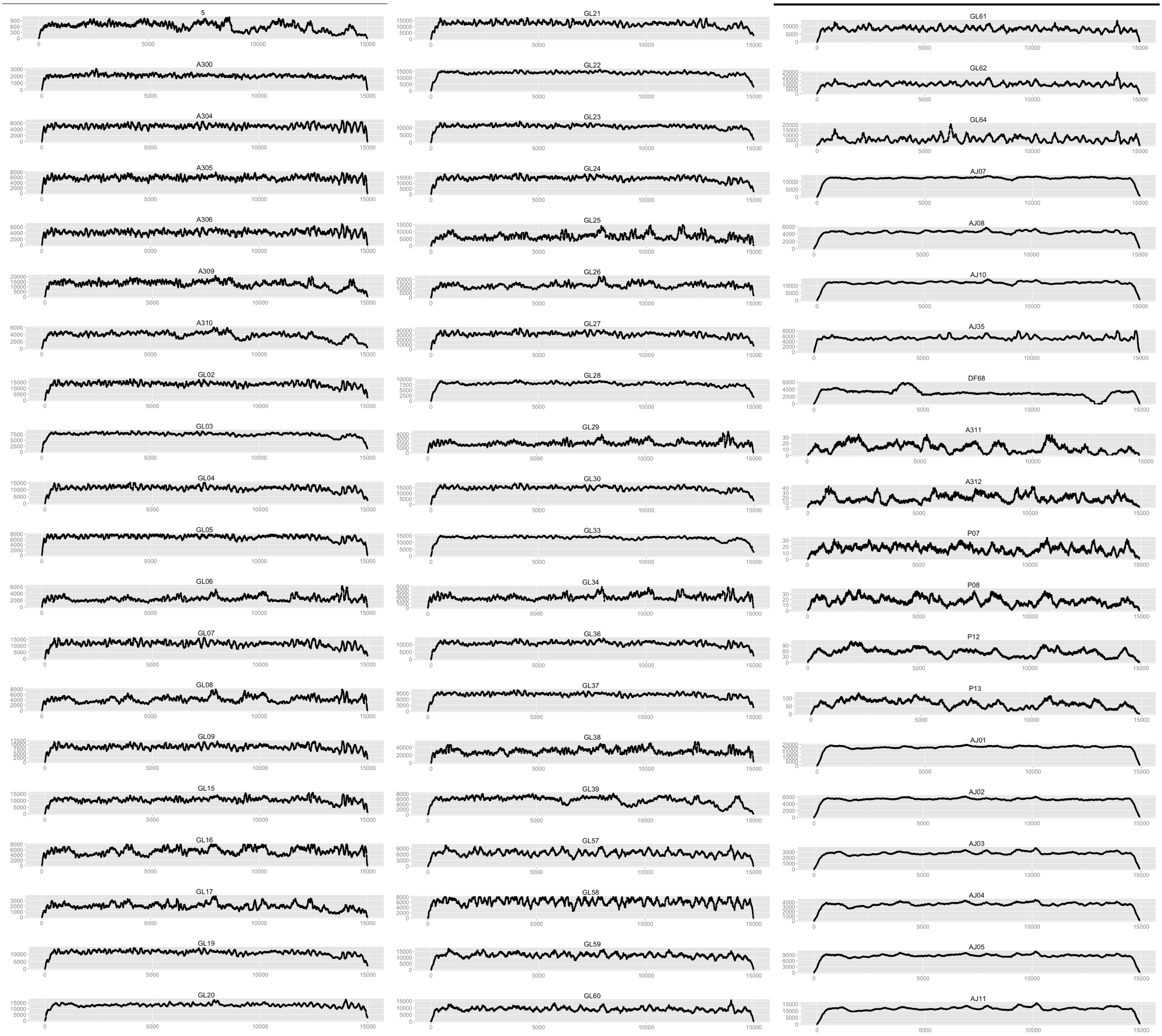

Supplementary Fig. S1. Coverage plots for 60 mitogenomes assembled for population analyses of the blowfly *C. megacephala* and the housefly *M. domestica*. All sequences were generated by WGS.

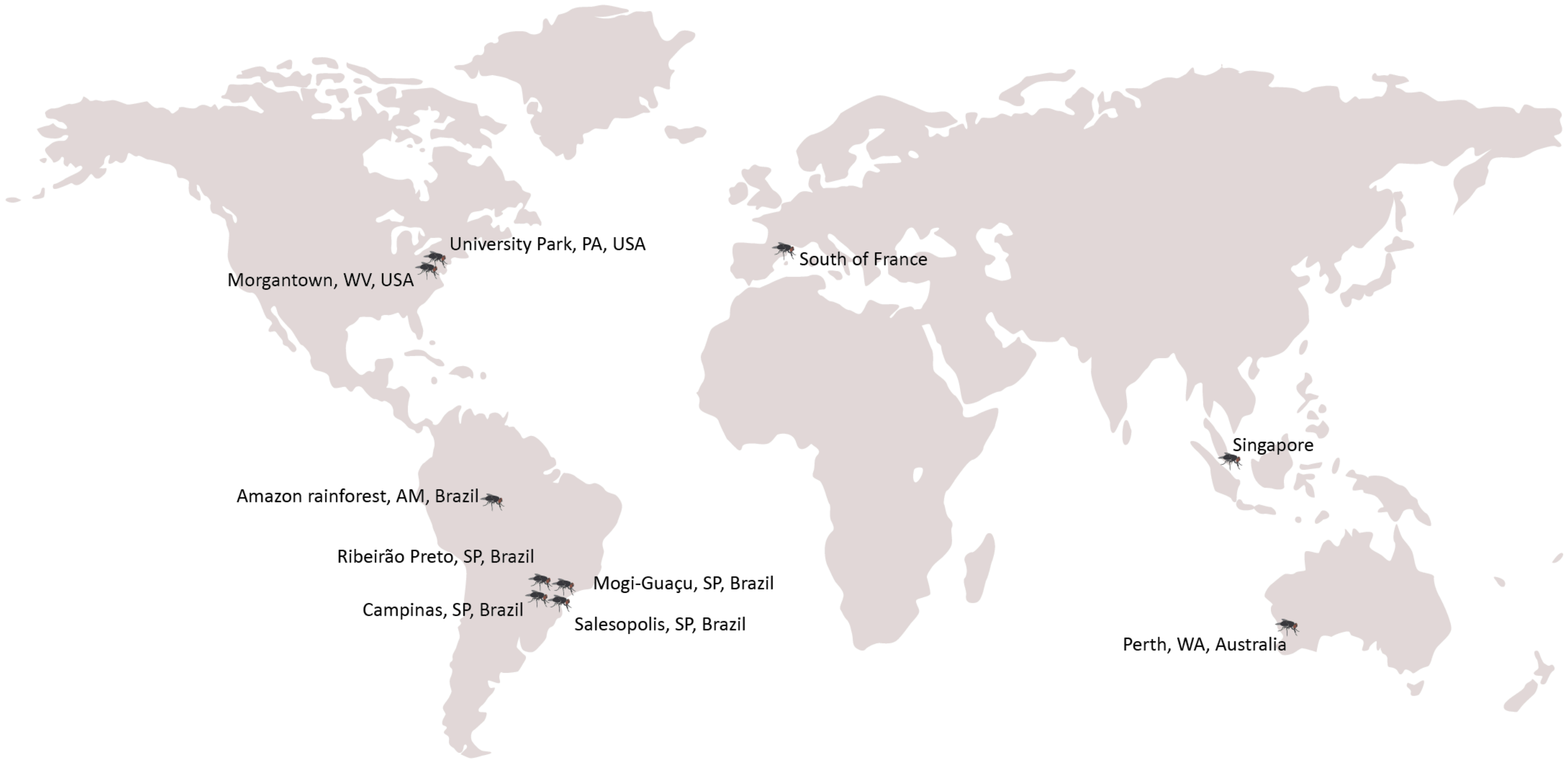

Supplementary Figure S2. Location of samples collected in this study. Samples from Campinas, Salesopolis, Mogi-Guaçu, Ribeirão Preto (SP, Brazil), Amazon (AM, Brazil), University Park (PA, USA), Perth (WA, Australia) and Singapore were freshly sampled, while fly specimens from Morgantown (WV, USA) and South of France were shipped by collaborators in absolute Ethanol. The full lists of samples with corresponding locations and geographical coordinates (for population analyses) are in Supplementary Tables S3 and S5. Map was adapted from original file available at Vector Graphics by VectorOpenStock.com, under Creative Common 3.0 Attribution License.

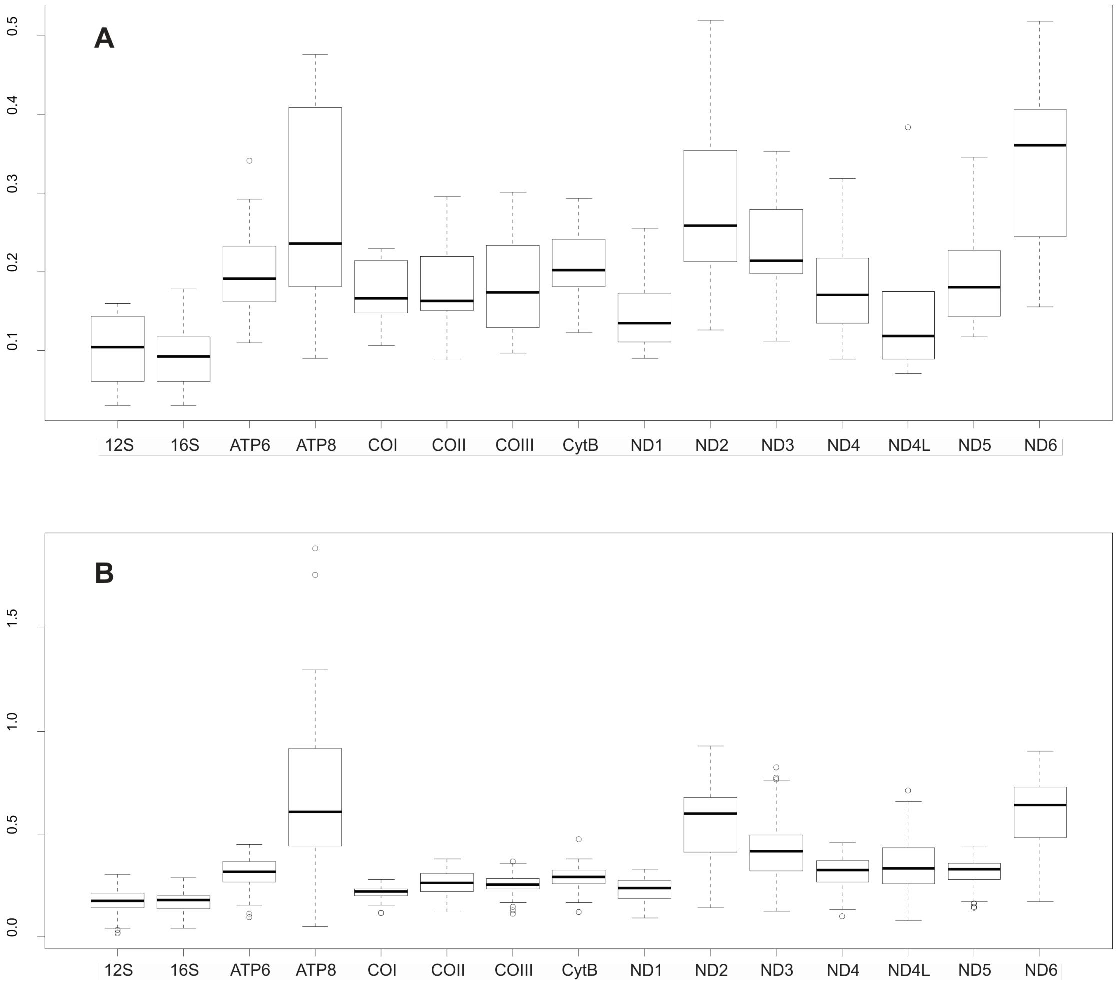

Supplementary Fig. S3. Box-plot graphics depicting the ranges of variation of genetic distances intra- (A) and inter- (B) groups (families). Genetic distances were estimated using a Maximum Composite Likelihood model with rate variation among sites (gamma distribution, shape parameter = 0.5).

A

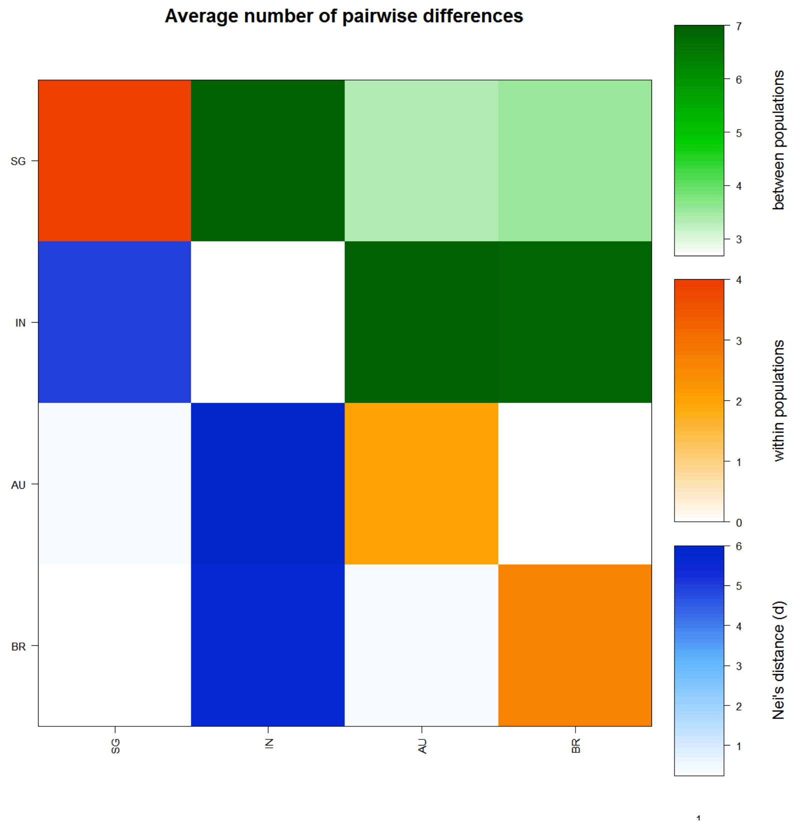

B

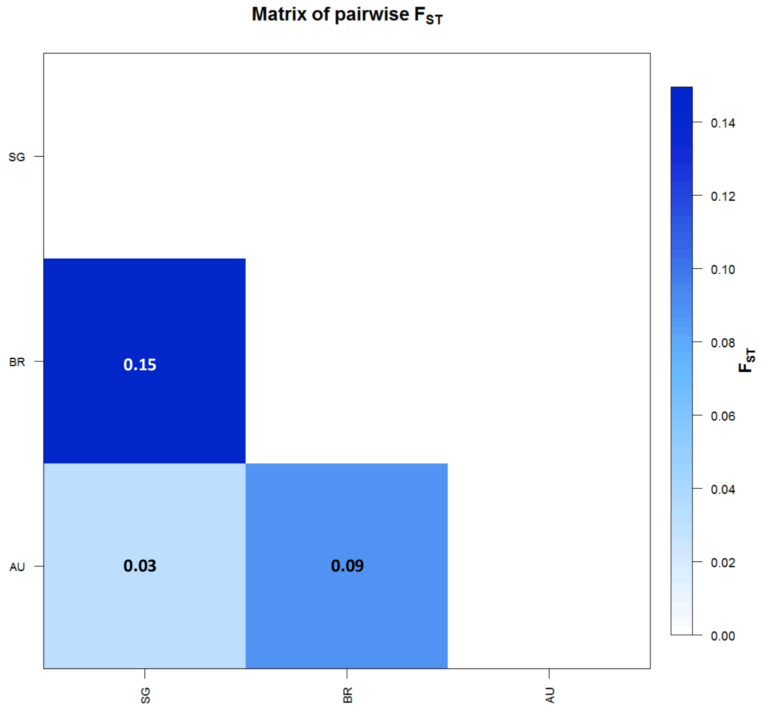

Supplementary Fig. S4. (**A**) The upper half of the matrix (green tones) shows the average number of pairwise differences between each sampled group of *C. megacephala* where AU = Australia, BR = Brazil, IN = India and SG = Singapore. The diagonal (orange tones) show the average number of pairwise differences within sampled continents, while the lower half (blue tones) shows the corrected average pairwise Nei's distance between the groups. The IN group is represented by the RefSeq sequence from India and therefore could represent a rare haplotype isolated from other populations. (**B**) Matrix of F_{ST} pairwise values between sub-groups mentioned above, showing low values among Brazilian, Australian and Singaporean samples. The RefSeq from India sub-group was excluded to minimize biases due to low number of samples (n=1).

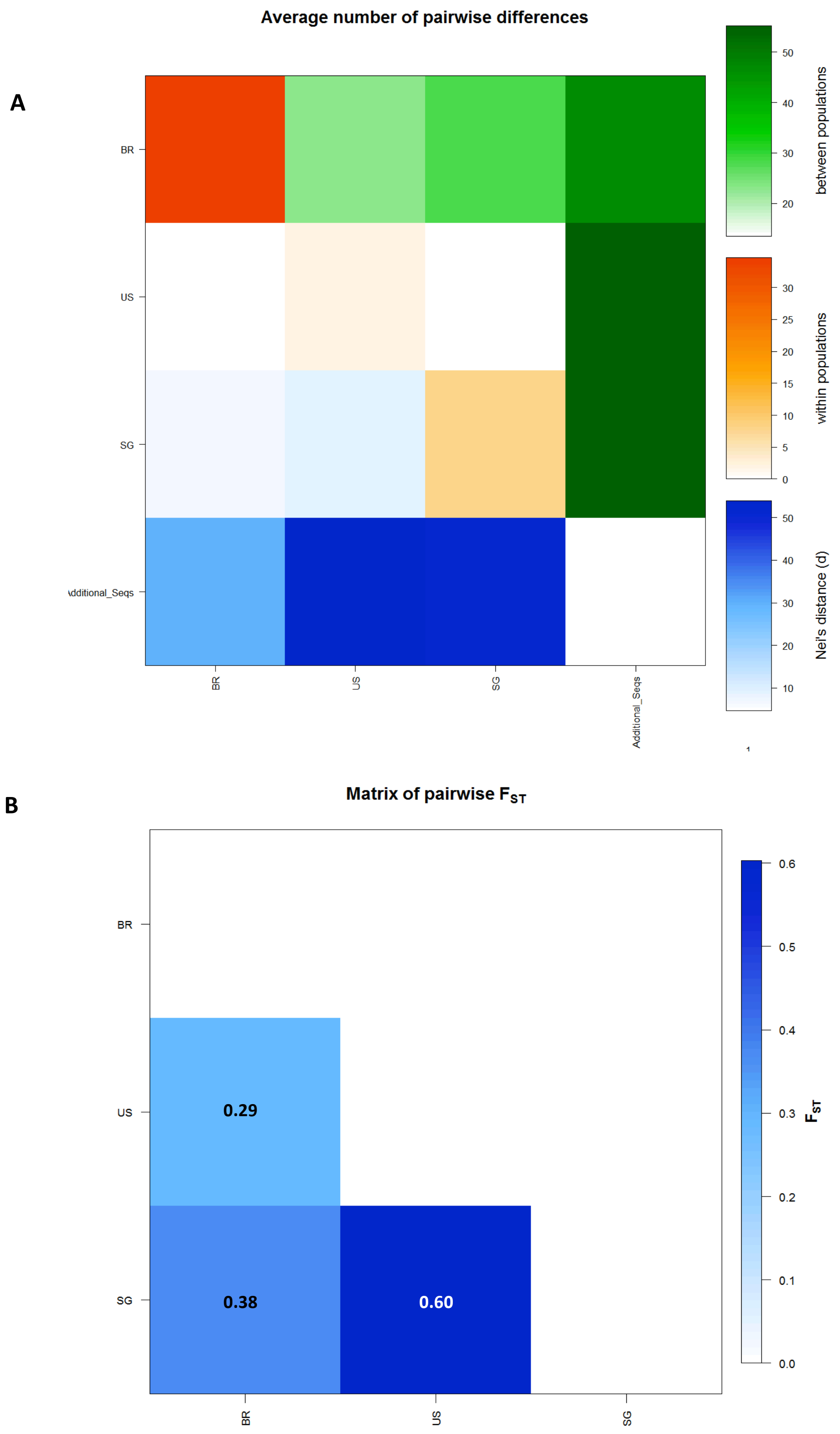

Supplementary Fig. S5. (**A**) The upper half of the matrix (green tones) shows the average number of pairwise differences between each sampled group of *M. domestica* where SG = Singapore, US = United States and BR = Brazil. The diagonal (orange tones) shows the average number of pairwise differences within sampled continents and the lower half (blue tones) shows the corrected average pairwise Nei's distance between the groups. "Additional_Seqs" refers to the RefSeq mtDNA of the housefly, for which no metadata is available. (**B**) Matrix of the F_{ST} pairwise between sub-groups mentioned above, showing higher values (dark blue) between the US and SG populations analyzed. The RefSeq sequence was removed to minimize biases due to low sampling (n=1).

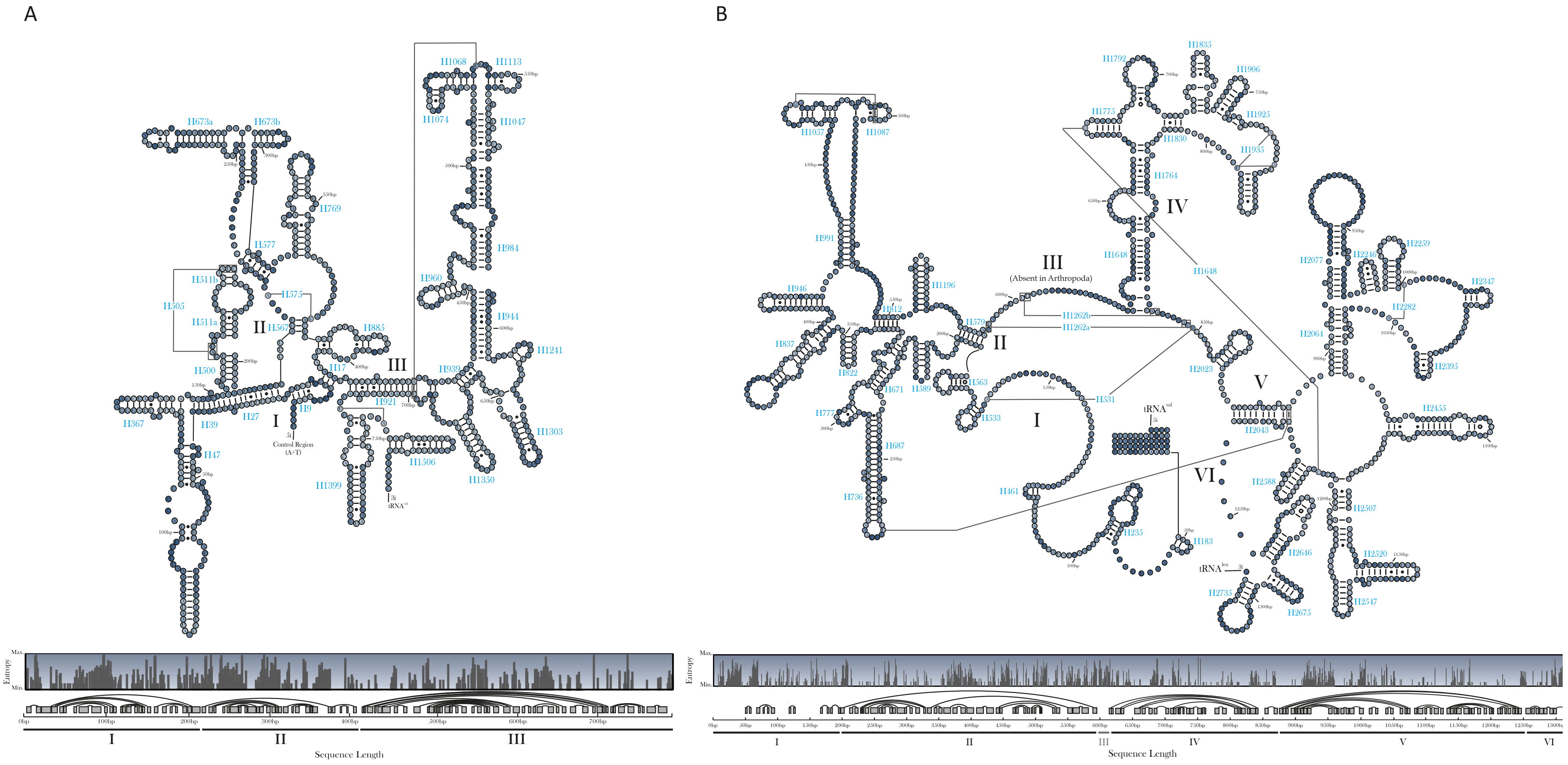

Supplementary Fig. S6. Inferred secondary structures of the 12S (**A**) and 16S (**B**) rRNAs subunits used as additional characters for Bayesian phylogenetic inferences. Helix names follow the *E. coli* system available at the CRW site. Positional variability in terms of entropy is below the structures. Color of the nucleotide sites indicates the positional variability found over 47 Schizophora species compared, where dark blue indicates high variable site and light blue indicates conserved regions in the molecule.

Supplementary Table S1. Amount of reads generated and mapped against the mtDNA reference used for assemblies of 32 different species of the Schizophora radiation. Coverage was based on the number of bases aligned to the total size of each mtDNA. The percentage of reads and bases aligned to the mtDNA reference likely corresponds to the mitochondrial fraction present in each library. As expected, long-range PCR libraries yield a higher amount of reads assigned to mtDNA when compared to WGS libraries that contain nuclear and metagenome reads as well. Average values are highlighted in grey.

Species	Method	Reads generated	Bases generated	Reads aligned	Reads aligned(%)	Bases aligned	Bases aligned(%)	Consensus bases	Coverage
Chloroprocta idioidea	PCR+shotgun	1,058,328	159,807,528	894,391	84.51	131,561,860	82.33	14,900	8830
Drosophila roehrae	PCR+shotgun	675,324	101,973,924	581,988	86.18	84,947,729	83.30	15,050	5644
Drosophila unipunctata	PCR+shotgun	495,018	74,747,718	435,681	88.01	63,956,463	85.56	15,091	4238
Drosophila mediopunctata	PCR+shotgun	536,136	80,956,536	453,346	84.56	66,269,701	81.86	14,993	4420
Sarcophaga bullata	PCR+shotgun	532,216	80,364,616	349,975	65.76	51,261,506	63.79	14,910	3438
Morellia lopesae	PCR+shotgun	280,826	42,404,726	254,881	90.76	37,274,289	87.90	14,960	2492
Mesembrinella sp.	PCR+shotgun	824,684	124,527,284	645,681	78.29	95,144,881	76.40	14,856	6404
Lucilia cuprina	PCR+shotgun	789,312	119,186,112	674,311	85.43	99,243,620	83.27	14,929	6648
Chrysomya albiceps	PCR+shotgun	563,890	85,147,390	447,833	79.42	66,228,386	77.78	14,947	4431
Marmarodeceia marmorata	PCR+shotgun	686,360	103,640,360	449,755	65.53	65,530,053	63.23	14,973	4377
Pseudogriphoneura sp.	PCR+shotgun	582,450	87,949,950	521,632	89.56	77,032,129	87.59	15,011	5132
Archisepsis discolor	PCR+shotgun	570,826	86,194,726	325,684	57.05	48,075,841	55.78	14,894	3228
Microsepsis sp.	PCR+shotgun	610,266	92,150,166	409,188	67.05	60,332,846	65.47	14,934	4040
Ocyptamus sativus	PCR+shotgun	462,384	69,819,984	377,131	81.56	55,297,792	79.20	15,206	3637
Musca domestica	PCR+shotgun	413,912	62,500,712	367,464	88.78	54,373,165	87.00	14,924	3643
Protophormia terranovae	PCR+shotgun	531,286	80,224,186	478,435	90.05	70,837,231	88.30	14,924	4747
	Average	600,826	90,724,745	479,211	80.16	70,460,468	78.05	14,969	4709
Chrysomya megacephala	WGS	72,627,600	7,335,387,600	749,837	1.03	75,081,702	1.02	14,985	5010
Cochliomyia macellaria	WGS	1,810,556	273,393,956	11,585	0.64	1,722,442	0.63	14,924	115
Paralucilia sp.	WGS	609,790	92,078,290	8,321	1.36	1,248,643	1.36	14,928	84
Hemilucilia sp.	WGS	710,974	107,357,074	8,036	1.13	1,199,914	1.12	14,907	80
Chrysomya putoria	WGS	695,276	104,986,676	4,955	0.71	742,493	0.71	14,985	50
Stomoxys calcitrans	WGS	516,462	77,985,762	11,758	2.28	1,762,436	2.26	14,930	118
Lucilia sericata	WGS	62,561,690	6,256,169,000	1,416,955	2.26	139,638,552	2.23	14,929	9354
Phormia regina	WGS	41,783,750	4,178,375,000	1,998,157	4.78	196,451,143	2.92	14,918	13169
Winthemia rufopicta	WGS	170,922	25,809,222	4,033	2.36	600,544	2.33	15,033	40
Boettcheria latisterna	WGS	1,235,196	183,355,923	1,843	0.15	267,888	0.15	14,944	18
Boettcheria bisetosa	WGS	1,179,624	176,163,873	7,253	0.61	1,067,814	0.61	14,944	71
Lucilia coeruleiviridis	WGS	1,009,248	150,380,502	16,506	1.64	2,417,915	1.61	14,989	161
Lucilia illustris	WGS	1,153,462	170,956,189	1,534	0.13	224,402	0.13	14,875	15
Eudasyphora canadiana	WGS	1,021,446	152,225,904	26,504	2.59	3,897,070	2.56	15,056	259
Hydrotaea sp.	WGS	1,066,548	158,312,284	11,552	1.08	1,699,044	1.07	15,112	112
Muscina levida	WGS	1,103,700	163,129,634	1,237	0.11	178,385	0.11	14,847	12
	Average	11,828,515	1,225,379,181	267,504	1.43	26,762,524	1.30	14,957	1792

Supplementary Table S2. List of samples used for population analyses, including 48 specimens of the blowfly *C. megacephala* and 13 specimens of the housefly *M. domestica*. The amount of reads generated and aligned against the mtDNA reference is shown, except for the sample F03 (*), which was assembled with a *de novo* pipeline (see Methods). The percentage of reads and bases likely corresponds to the mitochondrial fraction present in the libraries.

Sample	Reads generated	Bases generated	Reads aligned	Read aligned(%)	Bases aligned	Bases aligned(%)	Consensus bases	Coverage
Chrysomya megacephala								
L05	88,079,266	8,896,005,866	95,610	0.11	9,233,179	0.10	14,985	616
A300	80,365,128	8,116,877,928	307,043	0.38	30,828,927	0.38	14,985	2,057
A304	72,627,600	7,335,387,600	749,837	1.03	75,081,702	1.02	14,985	5,010
A305	90,397,490	9,130,146,490	879,851	0.97	87,876,119	0.96	14,985	5,864
A306	80,085,736	8,088,659,336	638,730	0.80	63,832,904	0.79	14,985	4,260
A309	79,536,408	8,063,656,208	2,067,624	2.60	204,810,315	2.54	14,985	13,668
A310	68,103,058	6,938,382,358	598,398	0.88	59,696,613	0.86	14,985	3,984
GL02	91,084,590	9,237,216,590	2,016,558	2.21	202,078,909	2.19	14,985	13,485
GL03	78,621,614	7,972,583,014	1,144,602	1.46	114,265,308	1.43	14,985	7,625
GL04	91,550,874	9,283,826,974	1,669,345	1.82	167,131,177	1.80	14,985	11,153
GL05	74,828,072	7,588,308,772	1,059,067	1.42	106,031,397	1.40	14,985	7,076
GL06	64,281,026	6,518,874,826	377,192	0.59	37,662,890	0.58	14,985	2,513
GL07	77,922,900	7,902,773,200	1,743,104	2.24	174,193,723	2.20	14,985	11,625
GL08	73,408,668	7,442,857,668	639,018	0.87	63,961,317	0.86	14,985	4,268
GL09	73,763,274	7,480,348,774	1,323,213	1.79	132,332,432	1.77	14,985	8,831
GL15	86,567,480	8,778,822,880	1,600,614	1.85	160,221,484	1.83	14,985	10,692
GL16	73,937,948	7,498,429,448	841,156	1.14	84,185,938	1.12	14,985	5,618
GL17	83,833,528	8,501,997,928	311,774	0.37	31,084,848	0.37	14,985	2,074
GL19	78,206,320	7,930,470,520	1,668,930	2.13	167,153,200	2.11	14,985	11,155
GL20	76,656,832	7,774,380,132	2,000,326	2.61	199,754,459	2.57	14,985	13,330
GL21	66,930,476	6,786,359,476	1,862,978	2.78	186,086,517	2.74	14,985	12,418
GL22	65,222,514	6,615,997,114	2,125,725	3.26	212,676,838	3.21	14,985	14,193
GL23	81,176,106	8,232,005,006	1,637,471	2.02	163,976,284	1.99	14,985	10,943
GL24	76,525,772	7,760,276,872	2,135,240	2.79	213,986,674	2.76	14,985	14,280
GL25	69,041,542	7,000,626,042	1,005,413	1.46	100,649,137	1.44	14,985	6,717
GL26	74,351,016	7,540,505,116	2,036,399	2.74	203,495,299	2.70	14,985	13,580
GL27	67,165,722	6,812,880,022	4,785,825	7.13	478,952,258	7.03	14,985	31,962
GL28	62,252,240	6,314,136,340	1,188,859	1.91	119,069,122	1.89	14,985	7,946
GL29	82,707,080	8,388,836,680	311,653	0.38	31,214,518	0.37	14,985	2,083
GL30	70,435,318	7,145,204,218	2,167,612	3.08	216,812,677	3.03	14,985	14,469
GL33	89,762,162	9,103,476,062	2,019,418	2.25	201,872,482	2.22	14,985	13,472
GL34	82,437,922	8,360,121,822	383,578	0.47	38,336,776	0.46	14,985	2,558
GL36	75,090,798	7,615,759,198	1,656,486	2.21	165,507,527	2.17	14,985	11,045
GL37	71,556,646	7,256,218,646	1,250,183	1.75	125,200,005	1.73	14,985	8,355
GL38	60,979,096	6,182,506,796	4,720,210	7.74	471,707,822	7.63	14,985	31,479
GL39	68,886,958	6,983,976,058	862,721	1.25	86,175,392	1.23	14,985	5,751
GL57	89,853,402	13,567,863,702	815,817	0.91	114,122,299	0.84	14,985	7,616
GL58	97,217,708	14,679,873,908	731,509	0.75	102,147,116	0.70	14,985	6,817
GL59	150,731,324	22,760,429,924	1,459,568	0.97	203,480,929	0.89	14,985	13,579
GL60	71,937,650	10,862,585,150	1,040,345	1.45	148,566,150	1.37	14,985	9,914
GL61	81,101,696	12,246,356,096	880,057	1.09	128,955,451	1.05	14,985	8,606
GL62	96,987,302	14,645,082,602	1,239,240	1.28	179,422,903	1.23	14,985	11,974
GL64	110,461,608	16,679,702,808	738,727	0.67	102,915,521	0.62	14,985	6,868
AJ07	91,798,374	23,041,391,874	767,103	0.84	187,968,451	0.82	14,985	12,544
AJ08	58,671,412	14,726,524,412	283,113	0.48	69,340,156	0.47	14,985	4,627
AJ10	134,538,450	33,769,150,950	739,939	0.55	181,309,808	0.54	14,985	12,099
AJ35	101,108,566	15,267,393,466	549,293	0.54	81,504,405	0.53	14,985	5,439
F03*	5,893,268	49,845,285,014	72,445	1.23	352,897,180	0.71	15,835	21,488
Musca domestica								
DF68	413,912	62,500,712	368,605	89.05	54,493,264	87.19	14,924	3,651
A311	633,034	95,588,134	1,298	0.21	193,307	0.20	14,924	13
A312	421,018	63,573,718	2,135	0.51	312,683	0.49	14,924	21
P07	80,274,132	8,164,916,979	122,448	0.15	12,183,067	0.15	14,924	816
P08	68,331,934	6,956,822,101	134,608	0.20	13,417,427	0.19	14,924	899
P12	56,904,330	5,790,258,632	402,307	0.71	40,179,214	0.69	14,924	2,692
P13	69,612,248	7,090,864,309	436,366	0.63	43,655,296	0.62	14,924	2,925
AJ01	104,038,638	26,113,698,138	1,341,861	1.29	329,441,248	1.26	14,924	22,075
AJ02	95,926,544	24,077,562,544	328,786	0.34	80,591,361	0.33	14,924	5,400
AJ03	79,638,430	19,989,245,930	173,902	0.22	42,497,166	0.21	14,924	2,848
AJ04	61,723,026	15,492,479,526	218,620	0.35	53,677,973	0.35	14,924	3,597
AJ05	104,727,800	26,286,677,800	535,151	0.51	131,413,184	0.50	14,924	8,805
AJ11	244,673,864	61,413,139,864	723,086	0.30	176,685,815	0.29	14,924	11,839

Supplementary Table S3. List of sequences of schizophoran species used for phylogenetic analyses and divergence time estimation. Mitogenomes access numbers provided by this work are in red. The * refers to the F03 sample assembled with long reads and used as a reference for further population studies. Near complete (partial) mtDNA genomes were reported by Wiegmann et al.[1]. Subfamilies of Calliphoridae were classified according to Rognes[2].

Subsection	Superfamily	Family	Subfamily	Species (sample ID)	Accession number	Location
-	Syrphoidea	Syrphidae	Syrphinae	*Ocyptamus sativus* (DF34)	KT272862	Ribeirão Preto, SP, Brazil
Acalyptratae	Opomyzoidea	Agromyzidae	Agromyzinae	*Liriomyza sativae*	NC_015926	
	Tephritoidea	Tephritidae	Dacinae	*Bactrocera oleae*	NC_005333	
				Ceratitis capitata	NC_000857	
	Sciomyzoidea	Sepsidae	Sepsinae	*Archisepsis discolor* (DF29)	KT272843	Ribeirão Preto, SP, Brazil
				Microsepsis sp. (DF30)	KT272774	Ribeirão Preto, SP, Brazil
	Lauxanioidea	Lauxaniidae	Lauxaniinae	*Pseudogriphoneura* sp. (DF28)	KT272855	Ribeirão Preto, SP, Brazil
				Marmarodeceia marmorata (DF20)	KT272847	Ribeirão Preto, SP, Brazil
	Ephydroidea	Drosophilidae	Drosophilinae	*Drosophila mediopunctata* (A282)	KT272856	Lab. Strain, Campinas-SP, Brazil
				Drosophila roehrae (A280)	KT272858	Lab. Strain, Campinas-SP, Brazil
				Drosophila unipunctata (A281)	KT272846	Lab. Strain, Campinas-SP, Brazil
				Drosophila melanogaster	NC_001709	
				Drosophila yakuba	NC_001322	
				Drosophila pseudoobscura	NC_018348	
Calyptratae	Oestroidea	Sarcophagidae	Sarcophaginae	*Sarcophaga bullata* (DF02)	KT272859	Morgantown, WV, USA
				Boettcheria latisterna (C18)	KT272848	
				Boettcheria bisetosa (D17)	KT272844	
				Sarcophaga impatiens	NC_017605	
		Calliphoridae	Crysomyinae	*Protophormia terraenovae* (DF71)	KT272776	South of France
				Phormia regina (H01A)	KT272842	
				Chrysomya putoria (GL11)	KT272850	Lab. Strain, Campinas, SP, Brazil
				Chrysomya megacephala (A304)	KT272775*	Lab. Strain, Campinas, SP, Brazil
				Chrysomya albiceps (DF17)	KT272864	Campinas, SP, Brazil
				Chrysomya rufifacies	NC_019634	
				Cochliomyia macellaria (A308)	KT272853	Lab. Strain, Campinas, SP, Brazil
				Cochliomyia hominivorax	NC_002660	
				Paralucilia sp. (A313)	KT272861	Campinas, SP, Brazil
				Hemilucilia sp. (A315)	KT272860	Campinas, SP, Brazil
				Chloroprocta idioidea (A251)	KT272777	Mogi Guaçu, SP, Brazil
			Luciliinae	*Lucilia coeruleiviridis* (C08)	KT272780	University Park, PA, USA
				Lucilia cuprina (DF16)	KT272779	Campinas, SP, Brazil
				Lucilia sericata (H15A)	KT272854	University Park, PA, USA
				Lucilia illustris (C05)	KT272845	University Park, PA, USA
			Mesembrinelinae	*Mesembrinella* sp.² (DF13)	KT272778	Salesópolis, SP, Brazil
			Calliphorinae	*Calliphora vicina*	NC_019639	
		Tachinidae	Dexiinae	*Rutilia goerlingiana*	NC_019640	
			Exoristinae	*Winthemia rufopicta* (S04A)	KT272849	University Park, PA, USA
		Oestridae	Cuterebrinae	*Dermatobia hominis*	NC_006378	
			Hypodermatinae	*Hypoderma lineatum*	NC_013932	
	Muscoidea	Muscidae	Muscinae	*Eudasyphora canadiana* (D02)	KT272852	University Park, PA, USA
				Musca domestica (DF68)	KT272840	Campinas, SP, Brazil
				Morellia lopesae (DF11)	KT272863	Salesópolis, SP, Brazil
				Stomoxys calcitrans (H04A)	KT272851	University Park, PA, USA
			Azelinae	*Hydrotaea* sp. (P10)	KT272841	University Park, PA, USA
				Muscina levida (D14)	KT272866	University Park, PA, USA
		Anthomyiidae	Anthomyiinae	*Delia radicum*	Partial	
		Scathophagidae	Scathophaginae	*Scathophaga stercoraria*	NC_024856	
	Hippoboscoidea	Glossinidae	N/A	*Glossina morsitans*	Partial	

Supplementary Table S4. Bayes factor model comparison analyses. Positive values indicate support for M_1 (row models) over M_0 (column models). All comparisons were significant (>200 in likelihood values), favoring the mtDNA11PF partitioning strategy (value in bold). Despite the similarity in topologies recovered, significant differences among partitioning strategies were also found, consistent with previous reports for Schizophora.

Maginal Log L (Harmonic Mean)	Partition Strategy		M0				
			mtDNA01	mtDNA05	mtDNA05PF	**mtDNA11PF**	mtDNA17
-183,363.76	M1	mtDNA01	-				
-180,929.58		mtDNA05	4,868.36	-			
-179,287.72		mtDNA05PF	8,152.08	3,283.72	-		
-170,961.97		**mtDNA11PF**	**24,803.58**	**19,935.22**	**16,651.50**	**-**	
-177,346.88		mtDNA17	12,033.76	7,165.40	3,881.68	**-12,769.82**	-

Supplementary Table S5. Samples used for population analyses of *C. megacephala* and *M. domestica*, including geographical coordinates of sampling sites and accession numbers for 64 samples used in the analyses (comprising samples available in GenBank). L05 sample was dried for 1 month before DNA extraction (1) and F03 is a pool of DNA extracted from 20 siblings used to generate long reads (2).

Species	Sample ID	Accession number	Location	Coordinates	
Chrysomya megacephala	A300	KT272811	Campinas, Sao Paulo, Brazil	22°54'13"S	47°01'08"W
	A304	KT272826		22°49'10"S	47°03'34"W
	A305	KT272804			
	A306	KT272812			
	L05[1]	KT272801			
	A309	KT272782			
	F03[2]	KT272775			
	A310	KT272783			
	GL02	KT272805		22°50'39"S	47°05'47"W
	GL03	KT272786			
	GL04	KT272791			
	GL05	KT272813			
	GL06	KT272814			
	GL07	KT272792			
	GL08	KT272824			
	GL09	KT272808			
	GL15	KT272815		22°54'57"S	47°08'50"W
	GL16	KT272816			
	GL17	KT272793			
	GL19	KT272799			
	GL20	KT272800			
	GL21	KT272802			
	GL22	KT272817			
	GL23	KT272825			
	GL24	KT272818			
	GL25	KT272803			
	GL26	KT272819			
	GL27	KT272794			
	GL28	KT272809			
	GL29	KT272820			
	GL30	KT272821			
	GL33	KT272822		22°49’38’’S	47°03’49’’W
	GL34	KT272785			
	GL36	KT272790			
	GL37	KT272810			
	GL38	KT272798			
	GL39	KT272789			
	GL57	KT272787	Manaus, AM, Brazil	3°5'47"S	59°59'24"W
	GL58	KT272788			
	GL59	KT272784			
	GL60	KT272806			
	GL61	KT272795			
	GL62	KT272807			
	GL64	KT272796			
	AJ07	KT272823	Singapore	1°20'39"N	103°40'48"E
	AJ08	KT272781			
	AJ10	KT272797			
	AJ35	KT272827	Gingin, WA, Australia	31°23'09.2"S	115°54'51.8"E
	GenBank	JX913738	Brisbane, QLD, Australia	NA	NA
	GenBank	AJ426041	Calicut, KL, India	NA	NA
Musca domestica	A311	KT272828	Campinas, SP, Brazil	22°49’38’’S	47°03’49’’W
	A312	KT272829		22°49’38’’S	47°03’49’’W
	DF68	KT272840		22°49'10"S	47°03'34"W
	P07	KT272830	University Park, PA, USA	40°48’52’’N	77°52’15’’W
	P08	KT272833			
	P12	KT272831			
	P13	KT272832			
	AJ01	KT272838	Singapore	1°20'42.3"N	103°40'45.3"E
	AJ02	KT272834	Singapore		
	AJ03	KT272839	Singapore		
	AJ04	KT272835	Singapore		
	AJ05	KT272837	Singapore		
	AJ11	KT272836	Singapore		
	GenBank	KM200723	NA	NA	NA

Supplementary Table S6. Estimation of costs per mitogenome sequence. Average estimation considers the last chemistry in sequencing and yielding for Illumina MiSeq (300x300 bp paired end run) and PacBio RSII (P6C4 chemistry). DNA extraction were estimated in ~USD3.00 per sample and long range PCR in ~USD4.00 per sample. Costs are included in the final price estimation, accordingly. The average percentage of bases aligned to the mtDNA reference were based on Supplementary Tables S1 and S2 (1.3% for MiSeq WGS, 78.05% for LR-PCR+MiSeq and 0.71% for PacBio RSII WGS) and used to calculate the mtDNA-related yield in each run. Maximum yield of sequencing runs were determined based on data generation at the sequencing facility at the Singapore Centre for Environmental Life Sciences Engineering.

	**MiSeq WGS**	**LR-PCR + MiSeq**	**PacBio RSII WGS**
**Maximum Yield**	12 Gb	12 Gb	0.7 Gb
**Read Length**	300x300 bp	300x300 bp	<10 kb
**Cost per run**	USD1500	USD1500	USD335
**Cost per library**	USD85	USD85	USD285
**Multiplexing**	Up to 96 samples	Up to 96 samples	Up to 10 samples*
**mtDNA yield**	195 Mb	9366 Mb	4.97 Mb
**Coverage**	101 X	6097 X	31 X
**Price per sample**	USD103	USD107	USD358

* Maximum number of multiplexed samples to allow minimum coverage of 30X.

Supplementary Table S7. Partitioning strategies evaluated for phylogenetic inferences under BI and ML methods. Best-fit selected substitution models for each alignment from near-complete mitochondrial genomes are shown. All PCGs were also divided by codon position and analyzed with PartitionFinder[3]. The rRNAs subunits were further partitioned by single (ss) and double (ds) stranded RNA sequence for BI analysis (mtDNA05 and mtDNA17).

Partitioning Strategy	Number of Partitions	Partition Scheme	Substitution Model
**mtDNA01** (By Genome)	**01**	All PCGs concatenated + 12S rRNA + 16S rRNA	GTR+I+G
**mtDNA03(05)** (By Gene)	**03(05)**	12S rRNA (ss)	HKY+I+G
		16S rRNA (ss)	GTR+I+G
		12S rRNA (ds), 16S rRNA (ds)	doublet[1]
		All PCGs concatenated	GTR+I+G
**mtDNA05PF** PartitionFinder (By Gene)	**05**	12S rRNA + 16S rRNA	GTR+I+G
		ND1 + ND4 + ND4L + ND5	GTR+I+G
		CO1 + CO2 + CO3 + CytB	GTR+I+G
		ATP6 + ND3 + ND6	GTR+I+G
		ATP8 + ND2	GTR+I+G
**mtDNA11PF** PartitionFinder (By Codon)	**11**	12S rRNA + 16S rRNA + 1st ATP8	GTR+I+G
		1st ND1 + 1st ND4 + 1st ND4L + 1st ND5	GTR+I+G
		2st ND1 + 2st ND4 + 2st ND4L + 2st ND5	GTR+I+G
		3st ND1 + 3st ND4 + 3st ND5	GTR+I+G
		1st ATP6 + 1st CO1 + 1st CO2 + 1st CO3 + 1st CytB	GTR+I+G
		2st ATP6 + 2st CO1 + 2st CO2 + 2st CO3 + 2st CytB + 2st ND3	GTR+I+G
		3st ATP6 + 3st ATP8 + 3st CO1 + 3st CO2 + 3st CO3 + 3st CytB + 3st ND3 + 3st ND6	GTR+I+G
		1st ND2 + 1st ND3 + 1st ND6	GTR+I+G
		2st ATP8 + 2st ND2 + 2st ND6	GTR+I+G
		3st ND4L	HKY+I+G
		3st ND2	HKY+I+G
**mtDNA15(17)** (By Gene)	**15(17)**	12S rRNA (ss)	HKY+I+G
		16S rRNA (ss)	GTR+I+G
		12S rRNA (ds), 16S rRNA (ds)	doublet[1]
		ND1, CytB, ND6, ND4L, ND4, ND5, ND3, CO3, ATP6, CO2, ND2	GTR+I+G
		ATP8, CO1	HKY+I+G

Supplementary Methods

Fossil constraints. Three fossil constraints were used for calibration, as following:

The Schizophora Crown

The origin of Schizophora clade likely occurred in the K-Pg boundary, with the main radiation taking place during the Tertiary[4]. The earliest record of the present Schizophora species is currently dated at the Paleocene (*Phytomyzites* spp), about 64 Ma[5]. In our analyses, a minimum age constraint of 64 Ma and a maximum age of 72 Ma (mid Cretaceous) were specified for Schizophora clade.

The Anthomyiidae

The oldest reliable record of a Calyptratae fly fossil, *Protanthomyia minuta,* is an anthomyiid fly preserved in Baltic amber and dated from the Eocene, about 42 Mya[6]. We applied a minimum age constraint at 42 Ma and a maximum age at 60 Ma, after the predicted Schizophora radiation.

The Oestroidea Crown

The oldest known Oestridae fly belongs to specimens from the Eocene shale of the Green River formation in North America. Biogeography data suggest that Oestroidea originated in the same period[7]. A minimum age constraint at 35 Ma and a maximum age at 60 Ma were used for Oestroidea clade node.

References

1. Wiegmann, B. M. et al. Episodic radiations in the fly tree of life. Proceedings of the National Academy of Sciences USA 108, 5690–5695 (2011).

2. Rognes, K. The Calliphoridae (Blowflies)(Diptera: Oestroidea) are not a monophyletic group. Cladistics 13, 27–66 (1997).

3. Lanfear, R., Calcott, B., Ho, S. Y. W. & Guindon, S. PartitionFinder: combined selection of partitioning schemes and substitution models for phylogenetic analyses. *Molecular Biology and Evolution* **29,** 1695–1701 (2012).

4. Grimaldi, D. A. & Engel, M. S. Evolution of the Insects. (Cambridge University Press, 2005).

5. Winkler, I. S., Labandeira, C. C., Wappler, T. & Wilf, P. Distinguishing Agromyzidae (Diptera) Leaf Mines in the Fossil Record: New Taxa from the Paleogene of North America and Germany and their Evolutionary Implications. Journal of Paleontology 84, 935–954 (2010).

6. Michelsen, V. Oldest authentic record of a fossil calyptrate fly (Diptera): a species of Anthomyiidae from early Coenozoic Baltic amber. Studia Dipterologica 7, 11–18 (2010).

7. Pape, T. The Oestrid Flies: Biology, Host-parasite Relationships, Impact and Management. (2006).
